# Phylogenomic discernments into Anaerolineaceae thermal adaptations and the proposal of a candidate genus *Mesolinea*

**DOI:** 10.3389/fmicb.2024.1349453

**Published:** 2024-02-29

**Authors:** Katherine Bedoya-Urrego, Juan F. Alzate

**Affiliations:** ^1^Centro Nacional de Secuenciación Genómica, Sede de Investigación Universitaria, Universidad de Antioquia, Medellín, Colombia; ^2^Departamento de Microbiología y Parasitología, Facultad de Medicina, Universidad de Antioquia, Medellín, Colombia

**Keywords:** Anaerolineaceae, Chloroflexota, MAGs, genome evolution, thermophiles, mesophiles, anaerobic digester

## Abstract

This study delves into the evolutionary history of Anaerolineaceae, a diverse bacterial family within the Chloroflexota phylum. Employing a multi-faceted approach, including phylogenetic analyses, genomic comparisons, and exploration of adaptive features, the research unveils novel insights into the family’s taxonomy and evolutionary dynamics. The investigation employs metagenome-assembled genomes (MAGs), emphasizing their prevalence in anaerobic environments. Notably, a novel mesophilic lineage, tentatively named *Mesolinea*, emerges within Anaerolineaceae, showcasing a distinctive genomic profile and apparent adaptation to a mesophilic lifestyle. The comprehensive genomic analyses shed light on the family’s complex evolutionary patterns, including the conservation of key operons in thermophiles, providing a foundation for understanding the diverse ecological roles and adaptive strategies of Anaerolineaceae members.

## Introduction

Anaerobic digestion is a widely employed biological treatment for sewage sludge, aiming to eliminate pathogenic microorganisms and transform organic matter into biogas, primarily methane and carbon dioxide (Barber et al., 2011; Bovio et al., 2019; Harirchi et al., 2022). This process relies on the synergistic interaction of four main microbial trophic groups: hydrolytic bacteria (hydrolysis phase), fermenting bacteria (fermentation phase), syntrophic bacteria (acetogenesis phase), and methanogenic archaea (methanogenesis phase) (Náthia-Neves et al., 2018).

Over the last decade, the phylum Chloroflexota has been consistently identified in anaerobic digesters (Petriglieri et al., 2018). Members of this phylum function as anaerobic phototrophs, contributing to inorganic CO_2_ fixation, as well as the oxidation of carbon monoxide and nitrite (Narsing Rao et al., 2022). Anaerolineaceae, a family within Chloroflexota, is notably abundant in full-scale anaerobic reactors (Bovio-Winkler et al., 2021). Members of the Anaerolineaceae family share physiological and morphological characteristics, such as filamentous morphology, anaerobic growth, and the ability to ferment carbohydrates or amino acids. Isolates from this family have been reported in anaerobic bioreactors in Colombia, Japan, Denmark, and the United States (Sekiguchi, 2003; Imachi et al., 2014; Sun et al., 2016; Petriglieri et al., 2018).

Metagenomic technologies have proven invaluable in identifying members of the Anaerolineaceae family, considering the challenges of isolating these bacteria *in vitro*. This approach has enabled the identification of thousands of metagenome-assembled genomes (MAGs) from microbial communities in various environments (Chen et al., 2021). Currently, MAGs have become a crucial strategy to enhance our understanding of the metabolic potential, microbial genomes, and evolution of uncultivated microorganisms. The retrieval of genomes is essential for achieving high-resolution phylogenies, identifying novel uncultured taxa (McIlroy et al., 2017), and analyzing the metabolic potentials of microbial communities in diverse environments.

In this study, we employed a combined metataxonomic and metagenomic approach to identify members and isolate metagenome-assembled genomes (MAGs) of the Anaerolineaceae family from industrial-scale anaerobic digesters in municipal wastewater treatment plants located in the Andean region of Colombia. Additionally, we explored genomic databases and identified other related Anaerolineaceae MAGs. By integrating genomic information from Anaerolineaceae reference genomes and MAGs, we formulated a well-supported hypothesis regarding the evolutionary history of the family. This approach enabled us to identify a novel lineage, resembling a new genus, among mesophilic members of the family. The evolutionary analysis also facilitated the comparison of general genomic features of Anaerolineaceae and the identification of a set of putative proteins present exclusively in the thermophilic members of the family.

## Materials and methods

### Biosolid samples and DNA extraction

Biosolid samples were collected from three wastewater treatment plants (WWTP) located in the Andean region of Colombia. The San Fernando WWTP treats 1.3 m^3^/s of municipal wastewater (houses and industry), serving a population of 700,000 inhabitants. This plant utilizes the upflow anaerobic sludge blanket (UASB) method for solid treatment. The Aguas Claras WWTP processes 5 m^3^/s of municipal wastewater and caters to a population of 2,200,000 inhabitants. Solid treatment involves anaerobic digestion and thermic drying. The Cañaveralejo WWTP handles 4 m^3^/s of municipal wastewater, serving a population of 2,600,000 inhabitants. The solids are treated using anaerobic digestion.

The study encompassed two biosolid samples from each wastewater treatment plant (WWTP), collected between 2017 and 2021 in different months. The investigation includes the reanalysis of previously published metagenomic data. Additionally, new shotgun metagenomic sequences were generated for two biosolid samples from Cañaveralejo and Aguas Claras WWTP, collected in 2021.

DNA extraction was performed with the Powermax^®^ Soil DNA Isolation Kit (QIAGEN, Venlo, The Netherlands) following the manufacturer’s instructions. DNA quantification was performed using a Nanodrop 2000 spectrophotometer (Thermo Scientific, MA, USA), and DNA concentrations were normalized to 30 ng/μL. Illumina libraries were prepared and sequenced reading 300 bp paired-end reads in a MiSeq instrument at Macrogen Inc. (Seoul, Republic of Korea), following the service provider recommendations.

### Metataxonomic analysis

The V3–V4 hypervariable regions of bacterial and archaeal 16S rRNA genes were amplified with the primers Bakt_341F (5′- CCTACGGGNGGCWGCAG-3′) and Bakt_805R (5′-GACTACHVGGGTATCTAATCC-3′). Quality filters (sequences with Q < 35 and < 200 bp) were applied using CUTADAPT software (Martin, 2011). The filtered sequences underwent processing with the mothur pipeline according to the MiSeq standard operating procedure (SOP) (Schloss et al., 2009). Clustering reads into operational taxonomic units (OTUs) was performed at a distance limit of 0.03. Data were normalized with the “totalgroup” method, and rare OTUs with fewer than 3 sequences were removed for downstream analyses. Taxonomic assignation was obtained with the SILVA database tool v138_1 (Quast et al., 2012). The R package ggplot2 was used for creating the graphical representation.

### Metagenome shotgun sequencing analyses and MAG isolation

Metagenomic shotgun sequencing was conducted on the Illumina Novaseq 6000 platform, generating 150-base paired-end reads at Macrogen, South Korea. The shotgun library was prepared using the TruSeq Nano DNA Kit (Illumina, CA, USA).

The reads were processed with CUTADAPT software to eliminate adapters and poor-quality reads (< Q30), utilizing the following parameters: -j 20 -q 30 -m 70 –max-n 0 (v 2.10) (Martin, 2011). Reads shorter than 70 bases or identified as singletons were excluded from subsequent analysis. For shotgun metagenome assembly, MetaSPADES version v3.14.1 was employed with specified flags -t 40 -m 160, testing k-mer lengths (-k) of 55, 77, and 99 bases (Bankevich et al., 2012).

Metagenome-assembled genome (MAG) isolation was carried out using the METABAT pipeline (Kang et al., 2019). In this process, the clean read dataset was mapped against the scaffolds assembled by METASPADES using BOWTIE2 (Langmead and Salzberg, 2012), and the resulting BAM file was further processed with SAMTOOLS (sort and index) (Li et al., 2009). The CheckM package (Parks et al., 2015) was utilized for the identification of bacterial MAGs. Those with a contamination score below 20% and an assembly size exceeding 400k bp were selected for further annotation and taxonomic assignment. The ANI strategy was applied to identify members of the Anaerolineaceae family within the selected MAGs.

### Genomic and phylogenomic analysis

Genomic descriptive statistics were calculated with a custom python script. Statistical comparisons such as Kruskal–Wallis test and box plot graphics were performed in the R environment.

Phylogenomic analysis of the Anaerolineaceae family’s evolutionary history utilized reference genomes from species deposited in the RefSeq database of GenBank:

GCA_000199675 *Anaerolinea thermophila* UNI-1, GCA_0010501952 *Anaerolinea thermolimosa* IMO-1, GCA_0010502152 *Bellilinea caldifistulae* GOMI-1, GCA_0010502352 *Longilinea arvoryzae* KOME-1, GCA_001050275 *Leptolinea tardivitalis* YMTK-2, GCA_001192795 *Flexilinea flocculi* TC1, GCA_001306035 *Levilinea saccharolytica* KIBI-1, GCA_001306115 *Ornatilinea apprima* P3M-1, GCA_001306145 *Thermanaerothrix daxensis* GNS1, GCA_003385075 *Pelolinea submarina* DSM 23923, GCA_003966975 *Pelolinea submarina* MO-CFX1, GCA_0065691852 *Litorilinea aerophila* ATCC BAA-2444, GCA_018435025 *Brevefilum fermentans* SL1-B42, GCA_900184705 *Brevefilum fermentans* CAMBI-1.

In addition to metagenome-assembled genomes (MAGs) derived from Colombian biosolid samples within the Anaerolineaceae family, a set of 470 Genomes/MAGs was downloaded from NCBI datasets. From this collection, five genomes (GCA_003499715, GCA_003445715, GCA_002417685, GCA_034430095, GCA_937858405) were selectively chosen based on their close relatedness to *Candidatus Mesolinea* for subsequent evolutionary and comparative genomic analyses. Using the DNADIFF tool and setting thresholds at 70% aligned genome and > 90% genome identity, we identified five MAGs as putative members of *Candidatus Mesolinea*. The dataset also includes genomes obtained from specific Colombian wastewater treatment plants, namely SANFERNANDO_UDEA04, CANAVERALEJO_UDEA05 (bin32), and AGUASCLARAS_UDEA06 (bin17). All genomes underwent annotation using the DFAST bacterial genome annotation pipeline.^1^

Using the SONIC PARANOID Software (Cosentino and Iwasaki, 2019), we identified a total of 1235 single-copy proteins present in all Anaerolineaceae RefSeq genomes. The respective coding sequences (CDSs) of these proteins were employed to search for homologs in all tested Anaerolineaceae genomes, with the CDSs from *Longilinea arvoryzae* KOME-1 (GCF_001050235.1) serving as the reference. A super matrix was constructed, encompassing the identified CDS sequences. This construction involved aligning each individual CDS with its homologous sequences using MAFFT program (Katoh and Standley, 2013), and then merging them employing the CATSEQUENCES program.^2^ In cases where some CDSs were not detected in all strains, gaps were employed to fill the alignment in the respective region.

The aligned sequences were utilized to infer a maximum likelihood tree using IQTREE2 software (Minh et al., 2020) with 5,000 bootstraps, employing the best model search for each partition CDS with the flags -m MFP+MERGE and -rcluster 10. The best-fit models, as determined by the Bayesian Information Criterion (BIC) (Lanfear et al., 2012, 2014), were: TPM2+F+I+G4TIM2+F+R3, GTR+F+R4: GTR+F+I+G4, GTR+F+R3: TIM3+F+R3: TVM+F+R4, GTR+F+R4, TPM2+F+G4, GTR+F+R3, TVM+F+I+G4, GTR+F+I+G4: TVM+F+R2: TVM+F+I+G4, GTR+F+R4, GTR+F+R4, GTR+F+R4: GTR+F+R3, GTR+F+R4, TIM3+F+I+G4, GTR+F+R4, GTR+F+R3, GTR+F+R3, GTR+F+R4, GTR+F+R4, TN+F+I, TVM+F+I+G4, GTR+F+R4, TIM2+F+R4, GTR+F+R4, GTR+F+R3, TVM+F, TIM2+F+R4, TIM3+F+I+G4, TIM2+F+I+G4, TIM3+F+I+G4: TPM2+F+R3, TIM2+F+R3, TPM3+F+G4, TVM+F+R3, TVM+F+I+G4: TIM3e+R3, GTR+F+R3, GTR+F+R4, TIM2e+G4, GTR+F+R3, GTR+F+R4, K2P+I, GTR+F+R3, HKY+F+G4, GTR+F+R3, TPM3+F+R2. Thermophiles of the Caldilineae family, species *Caldilinea aerophila* and *Litorilinea aerophile*, were selected as outgroups for rooting the tree. The resultant phylogenetic tree was visualized and edited with the FigTree software.

### Genome and proteome conservation analysis

Genomic alignment and comparative analysis utilized the DNADIFF tool within the MUMMER v4 software (Marçais et al., 2018). Alignment was conducted for all Anaerolineaceae genomes, and key metrics, such as the fraction of aligned bases and average nucleotide identity, were extracted from the corresponding “.report” file. Subsequently, a non-redundant table was generated and imported into R for further analysis.

The Average Amino Acid Identity (AAI) score was computed using the EzAAI program (Kim et al., 2021). In this process, putative proteomes annotated with DFAST pipeline (Tanizawa et al., 2016) served as input for the comparisons. An exhaustive all-vs-all analysis of single-copy proteomes was executed, resulting in a non-redundant table containing AAI score values and proteome coverage ratios for all comparisons. This table was then used for subsequent statistical and graphical analyses in R, employing the ggplot2 library.

Orthologous analysis, such as the identification of thermophilic-specific proteins, was performed based on the results obtained with SONIC PARANOID tool (Cosentino and Iwasaki, 2019). Manual curation and annotation of the Mrp, Mox, sHPD, and SufE gene clusters were conducted using the ARTEMIS genome annotation tool (Carver et al., 2012).

## Results

### Novel Anaerolineaceae: a dominant taxon in sewage sludge from Colombian WWTPs

This study focused on the Anaerolineaceae family, consistently identified as one of the dominant taxa in sewage sludge (biosolids) from three anaerobic digesters treating municipal wastewater in the Andean region of Colombia (San Fernando, Aguas Claras, and Cañaveralejo). Previous metagenomic analyses allowed us to isolate a metagenome-assembled genome (MAG) of the dominant Anaerolineaceae in the sewage sludge of another WWTP, San Fernando, as reported in 2020 (GCA_008635265) (Benavides et al., 2020). For Aguas Claras and Cañaveralejo WWTPs, new shotgun metagenomic sequencing data were generated and analyzed.

**FIGURE 1:**
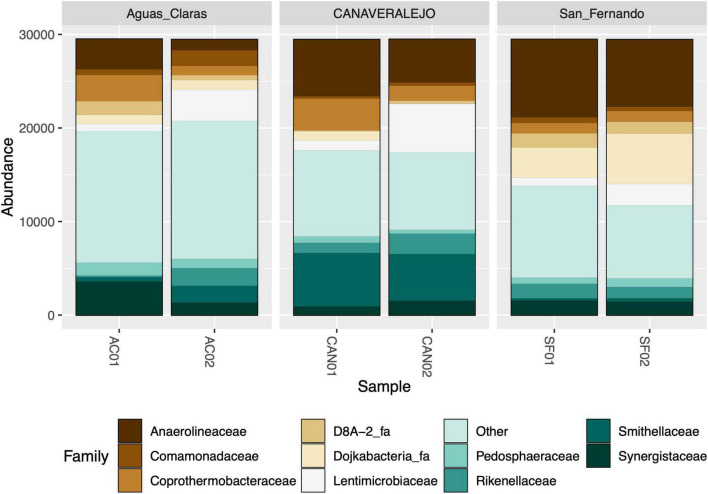
Stacked bar graph illustrating the relative abundance of bacterial families in three biosolid samples at two distinct time points. Aguas Claras (Antioquia), San Fernando (Antioquia), and Canaveralejo (Valle) WWTPs were sampled in two different months, and the relative abundance of high-quality amplicons is presented as normalized counts for the top ten most abundant bacterial families.

As an initial step, we conducted bacterial 16S rRNA gene metataxonomic analyses for three Andean Colombian WWTPs, confirming that Anaerolineaceae consistently ranks within the top ten most dominant bacterial families (Figure 1). These analyses were conducted in different months to assess the stability of Anaerolineaceae’s high abundance over time.

**FIGURE 2:**
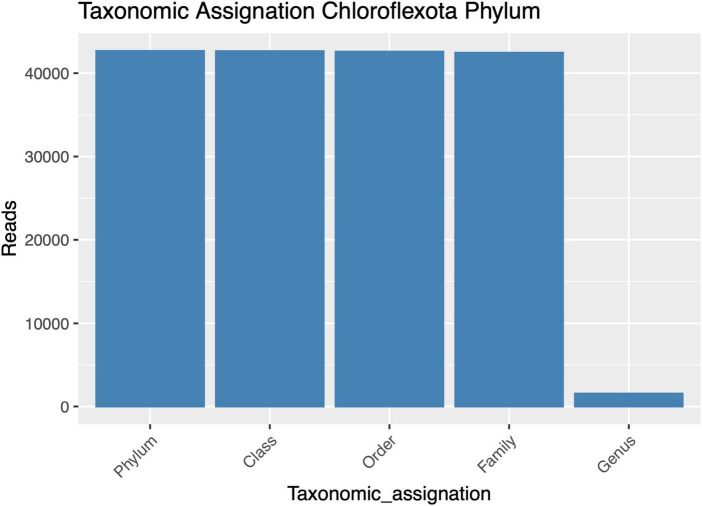
Taxonomic assignment success for high-quality amplicons of the Chloroflexota phylum, from phylum to genus. The *y*-axis presents the numbers of sequences assigned to each taxonomic category.

**FIGURE 3:**
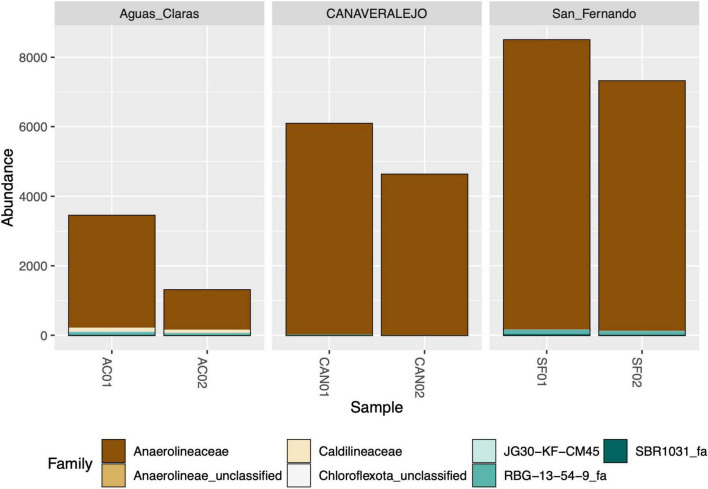
Stacked bar graph depicting the relative abundance of bacterial families within the Chloroflexota phylum in three biosolid samples at two distinct time points. Aguas Claras (Antioquia), San Fernando (Antioquia), and Canaveralejo (Valle) WWTPs were sampled in two different months, and the normalized counts of high-quality amplicons are presented for the top ten most abundant bacterial families within the Chloroflexota phylum.

Despite successfully identifying the 16S rRNA gene amplicons belonging to Anaerolineaceae, the assignment of these sequences to the genus category was largely unsuccessful, with most of the metagenomic operational taxonomic units (mOTUs) of this family lacking a genus assignment (Figure 2). Taxonomic assignment of the 16S rRNA gene sequences of Chloroflexota to the Family category performed well but sharply declined at the genus category, suggesting a microbiological novelty in the Anaerolineaceae family within these samples, not present in the Silva database. Additionally, we conducted a taxonomic analysis focused on the families of Chloroflexota present in the Andean biosolid samples, and even though Caldilineaceae is present, Anaerolineaceae is, by far, the dominant group (Figure 3).

### Metagenome-assembled genomes (MAGs) of Anaerolineaceae from the Andean plants of Aguas Claras and Cañaveralejo

Two independent shotgun metagenomic experiments were conducted, one for each biosolid sample from the two WWTPs, generating 11.8 Gb (77,955,936 reads) of raw read data for Aguas Claras and 12.6 Gb (83,354,128 reads) for Cañaveralejo. In both shotgun DNA-seq experiments, over 92% of raw read bases achieved a quality value of at least Q30. After quality trimming, approximately 98% of the initial shotgun reads were included in downstream analyses. Metagenome assembly using METASPAdes resulted in 910,400 and 1,528,506 scaffolds for Aguas Claras and Cañaveralejo, respectively. The largest contig encompassed 686,277 and 868,999 bp, respectively. The percentage of ambiguous bases (“Ns”) was below 0.5% for both assembled datasets.

The metagenomes were binned using MetaBAT2, generating between 86 and 140 bins per sample. These bins were analyzed using CheckM software for taxonomic assignment and to assess genome completeness and contamination. Most bins were assigned to the Bacteria kingdom, constituting 52 and 65% in Aguas Claras and Cañaveralejo biosolids, respectively. Phyla identified by CheckM included Proteobacteria, Firmicutes, Bacteroidetes, and Actinobacteria. Euryarchaeota was also identified but in lesser proportion (Supplementary Table 1).

Members belonging to the Chloroflexota phylum were not identified using the CheckM strategy. Acknowledging this limitation of CheckM, we conducted BLASTN searches against the assembled bins using the genome of Anaerolineaceae previously assembled from the San Fernando WWTP (GCA_008635265) as the query. Bins with positive hits were further confirmed with the phylogenetic analysis described below. Annotation analysis with the DFAST software revealed that genome contamination in both bins (MAGs) was below 1.5%. The same DFAST pipeline was employed to classify the Anaerolineaceae MAGs using the average nucleotide identity (ANI) strategy, and its results confirmed their relatedness to the Anaerolineaceae. Interestingly, this analysis also allowed us to identify other previously published MAGs closely related to the Colombian Anaerolineaceae MAGs (ANI > 85%) that were included in the downstream analysis.

### Evolutionary history of the Anaerolineaceae family

Genome sequences of 13 members of the Anaerolineaceae family, available in the NCBI-RefSeq database, were downloaded and analyzed alongside three Anaerolineaceae metagenome-assembled genomes (MAGs) isolated from Colombian Andean WWTPs (SANFERNANDO_UDEA04, CANAVERALEJO_UDEA05 _bin32, and AGUASCLARAS_UDEA06_bin17). The included genera with reference RefSeq genomes were *Anaerolinea*, *Bellilinea*, *Longilinea*, *Leptolinea*, *Flexilinea*, *Levilinea*, *Ornatilinea*, *Pelolinea*, *Brevefilum*, and *Thermanaerothrix*. Moreover, we incorporated five Anaerolineaceae MAGs from the GenBank datasets database into the phylogenetic analyses. These MAGs were chosen for their close relation, meeting the criteria of nucleotide identity surpassing 90%, with at least 70% of their genomes successfully aligned against the San Fernando Anaerolineaceae MAG (GCA_008635265). The included MAGs are as follows: GCA003445715, GCA002417685, GCA003499715, GCA_034430095, and GCA_937858405.

The phylogenomic strategy, based on single-copy orthologous protein-coding genes, successfully reconstructed the evolutionary history of Anaerolineaceae with robust support. A total of 1,235 coding DNA sequences (CDSs) were included in the alignment super matrix used for this phylogenetic reconstruction.

**FIGURE 4:**
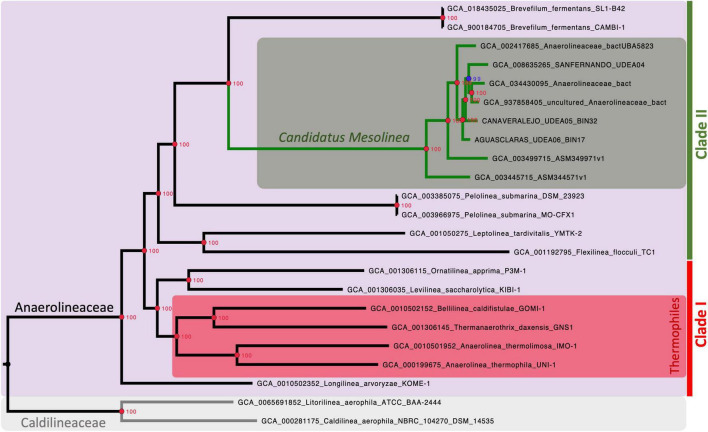
Maximum-likelihood phylogenomic tree illustrating the evolutionary relationships within the Anaerolineaceae family. The analysis is based on the alignment of 1,235 protein-coding sequences (CDSs). Caldilineaceae is used as the outgroup for comparison. Branch lengths represent genetic distances, and nodes indicate the divergence points along with the corresponding ultrafast bootstrap (UFB) support values. Red nodes denote 100% UFB support. Thermophilic members of the family are identified with a red rectangle. *Candidatus* nov. genus *Mesolinea* is highlighted with a green rectangle. Clades I and II of Anaerolineaceae are also explicitly labeled.

Our analysis strongly supports the monophyletic nature of the Anaerolineaceae family (UFB = 100%), with *Longilinea arvoryzae* KOME-1 identified as the most ancestral species. The family branches into two major clades: one encompassing *Levilinea*, *Ornatilinea*, *Anaerolinea*, *Thermanaerotrix*, and *Bellilinea*; and the other consisting of the genera *Flexilinea*, *Leptolinea*, *Pelolinea*, and *Brevefilum*. In cases where multiple reference genomes were available for a genus, well-supported monophyletic clades were formed with 100% UFB support (Figure 4).

The phylogenetic analysis unveils a striking pattern in the evolutionary history of the Anaerolineaceae family, particularly with the emergence of a distinct and strongly supported clade. This clade is exclusive to thermophilic members of the family and encompasses notable genera such as *Anaerolinea*, *Thermanerothrix*, and *Bellilinea*. A closer examination of the genomic characteristics within this thermophilic clade unveils a consistent elevation in GC content, accompanied by larger genome sizes spanning the range of 3.0 to 4.1 Mb.

In sharp contrast, the remaining Anaerolineaceae members, classified as mesophilic, exhibit lower median values of GC content. The upcoming sections will delve into this matter in more detail.

Interestingly, our novel Anaerolineaceae metagenome-assembled genomes (MAGs) from Colombian sewage sludge, alongside other MAGs from wastewater treatment systems in North America and Asia, constitute an independent and well-supported lineage within Anaerolineaceae. Positioned adjacent to the *Brevefilum* genus, these novel Anaerolineaceae MAGs form a robust monophyletic clade (Figure 4). Given their cohesive phylogenetic placement, these novel Anaerolineaceae MAGs will be treated as a group for downstream comparative genomic analyses, equivalent to a genus level.

### Genomic profiles in Anaerolineaceae

**FIGURE 5:**
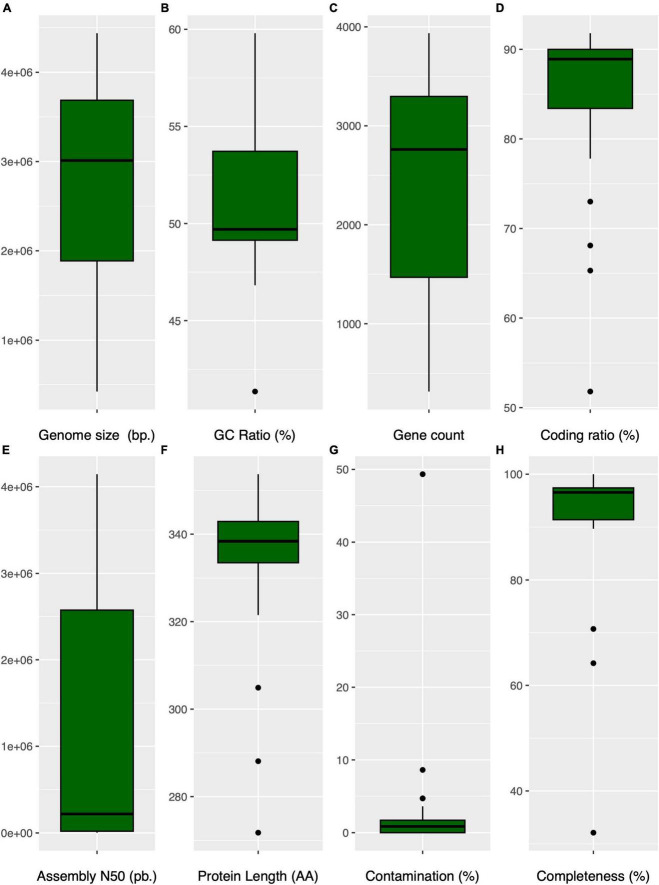
Comparative box plots illustrating genomic characteristics in Anaerolineaceae: **(A)** (genome size): Distribution of genome sizes (bp). **(B)** (GC content): Variation in GC content (%). **(C)** (gene count): Box plot depicting the distribution of gene counts per genome. **(D)** (coding ratio): Genomic coding percentage (%). **(E)** (assembly N50): Distribution of the assembly N50 values (bp.) for each genome. **(F)** (protein average length): Representation of the protein median length in amino acids per genome. **(G)** (contamination): Variation in genome contamination as assessed by CHECKM for each genome. **(H)** (genome completeness): Box plot showing the genome completeness (%) as assessed by CHECKM for each genome.

An overview of the general genomic characteristics of Anaerolineaceae genomes is presented in Supplementary Table 2. The genome sizes within the Anaerolineaceae family varied from 2.6 to 4.4 Mb, with a median size of 3,510,630 bp (Figure 5A). Notably, many of the novel Anaerolineaceae metagenome-assembled genomes (MAGs), including those from the Andean region, were relatively small, falling within the range of 1 to 2 Mb. However, the Cañaveralejo MAG (CANAVERALEJO_UDEA05_bin32) appeared unusually small at 423k, indicating severe genome incompleteness.

The genus *Brevefilum* exhibited the smallest genomes, approximately 2.7 Mb, while *Longilinea arvoryzae* KOME-1 possessed the largest genome, exceeding 4.4 Mb.

Regarding genomic GC content, the median value was 49.7%, displaying a broad dispersion ranging from 41.4% (*Flexilinea flocculi* TC1) to 59.8% (*Levilinea saccharolytica* KIBI-1) (Figure 5B). The Anaerolineaceae MAGs from the Andean region closely aligned with the median GC content, exhibiting a value of 49.5%.

The gene count per genome and the coding ratio exhibited median values of 2,761 genes and 89%, respectively. *Longilinea arvoryzae* KOME-1 displayed the highest gene count at 3,936, consistent with its larger genome size (Figures 5C, D). Notably, only three Anaerolineaceae genomes are reported as complete according to RefSeq records: *Anaerolinea thermophila* UNI-1, *Pelolinea submarina* MO-CFX1, and *Brevefilum fermentans* CAMBI-1, indicating that their chromosomes are represented in a single scaffold. The family’s median assembly N50-value is 219,703 bp (Figure 5E). The average protein length showed a relatively narrow dispersion range around its median value of 338 amino acids. However, the San Fernando Anaerolineaceae MAG displayed a notably lower value in this feature at 272 (Figure 5F). The variations in genome size, GC content, and other genomic features within the Anaerolineaceae family suggest an intricate interplay between genetic adaptations and environmental conditions. The smaller genomes and lower GC content observed in certain MAGs, particularly from the clade of the Andean region and *Brevefillum*, may reflect adaptations to specific ecological niches, possibly through gene losses and genome deletions. However, it is crucial to acknowledge that smaller genome sizes in MAGs could be attributed to limitations inherent in metagenomic sequencing and assembly technologies. The Assembly size in a MAG should not be interpreted as the actual genome size, as it may be underestimated due to inherent challenges such as incomplete assembly or loss of genomic content. The Cañaveralejo MAG (CANAVERALEJO_UDEA05_bin32), appearing unusually small at 423 kbp, underscores the importance of considering incompleteness and assembly artifacts. Therefore, caution should be exercised in directly correlating assembly size with true genome size, as the latter may indeed be larger, and the observed variations could be influenced by short read metagenomic sequencing and assembly limitations.

CheckM quality metrics indicated low contamination for most Anaerolineaceae genomes, generally below 5%, except for the San Fernando MAG (SANFERNANDO_UDEA04), which exhibited a value of 49.3%. Additionally, the genome completeness metric calculated with CheckM revealed that RefSeq reference genomes surpassed 95%, while the novel Anaerolineaceae MAGs exhibited lower values ranging from 92.8 to 32.1% (Figures 5G, H).

### Genome and proteome conservation in Anaerolineaceae

**FIGURE 6:**
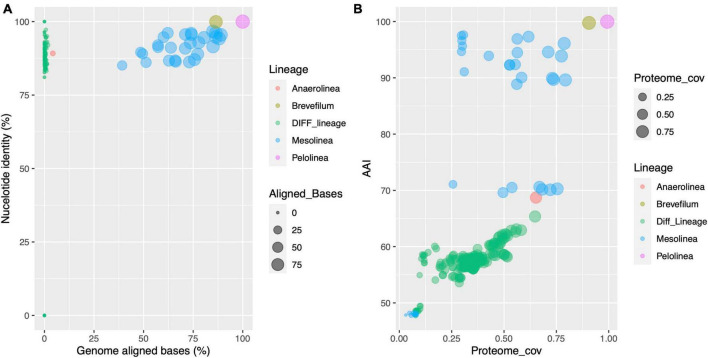
Scatter plot analysis of genome and proteome conservation in Anaerolineaceae. **(A)** Correspondence between genome nucleotide identity and the proportion of genome-to-genome aligned bases across different species of Anaerolineaceae. Each data point represents a pairwise comparison between two genomes. The *x*-axis depicts the proportion of genome-aligned bases, and the *y*-axis represents nucleotide identity. The color code distinguishes comparisons within the genus (whenever at least two genomes of the same genus were available) or inter-genus comparisons (green dots, label “Diff_Lineage”). **(B)** This scatter plot displays the correspondence between average amino acid identity (AAI) and the coverage of the putative proteome across different species of Anaerolineaceae. Each data point symbolizes a comparison between two species. The *x*-axis represents the proteome coverage ratio, while the *y*-axis represents average amino acid identity (AAI, percentage). The color code distinguishes comparisons within the genus (whenever at least two species of the same genus were available) or comparisons between different genera (green dots, label “Diff_Lineage”).

To gain insights into the genome and proteome conservation in Anaerolineaceae, comprehensive comparisons were conducted. Genome comparisons using the DNADIFF tool revealed low conservation of nucleotide sequences, with a median value of aligned genome bases at 0.2%. In contrast, comparisons within the same genus exhibited a significantly higher median value, exceeding 73%. Notably, the two *Pelolinea* isolates demonstrated remarkable similarity, with 99.97% of their genomes aligned and a global genome identity of 99.99%. These findings raise the possibility of redundant genome accessions in the NCBI database for the *Pelolinea* genus, where two separate genome sequences may pertain to the same isolate. Regarding the *Brevefilum* species, a substantial portion of their genomes aligned (86.5%), resulting in an overall nucleotide identity of 99.9% (Figure 6A).

On the other side of these comparisons are the two *Anaerolinea* reference species. Despite both being sister species of the same genus, only 4.2% of their genomes can be aligned, and within these regions, the average nucleotide identity reached 89.2%.

In the case of the novel Anaerolineaceae MAGs, they showed to be closely related, aligning between 39 and 89% of their genomes, and their nucleotide identity ranged from 85 to 97%.

The proteomes showed a more conserved profile, with the median value of average amino acid identity reaching 57.7% among the Anaerolineaceae proteomes, and the median value for proteome coverage ratio was 0.35 (35%). Again, when the comparison was performed, where possible, within their respective genera, the values rose to 90% and 0.53 (53%) for AAI and the proteome coverage ratio, respectively (Figure 6B).

### Distinctive genomic features in Anaerolineaceae clades

**FIGURE 7:**
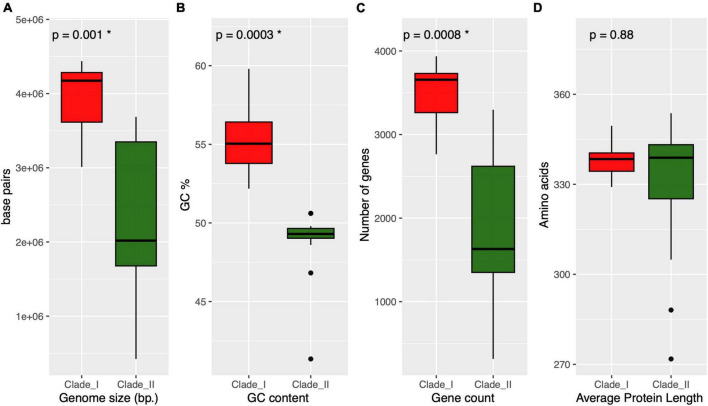
Overview of the genomic and proteomic characteristics across different clades of Anaerolineaceae. Each panel represents a specific parameter, and clades are differentiated by red and dark green colors. **(A)** (genome size): Boxplot representation of genome sizes, with statistical significance denoted by *p* = 0.001 (Kruskal–Wallis test). **(B)** (GC content): Boxplot presentation of GC content percentages, highlighting statistical significance with *p* = 0.0003 (Kruskal–Wallis test). **(C)** (gene count): Distribution of gene counts within clades, with significant differences marked by *p* = 0.0008 (Kruskal–Wallis test). **(D)** (average protein length): Boxplot illustration of average protein lengths, showing no statistical significance (*p* = 0.88, Kruskal–Wallis test). * Next to the *p*-value indicates statistical significance.

As depicted in the Anaerolineaceae phylogenomic tree, the family is divided into two well-supported clades. We sought to compare general genomic features among these two groups, including genome assembly size, GC content, gene count, and average protein length. As shown in the boxplots presented in Figure 7, the median values of the first three variables were significantly lower (Kruskal–Wallis rank sum test) in Clade II of Anaerolineaceae (Figures 7A–C). The median genome size value drops from 4.2 Mb in Clade I to 2 Mb in Clade II. The median number of genes per genome followed a similar trend, with lower counts in Clade II (1,630 genes) compared to Clade I (3,656 genes). The median GC content value also showed a differential profile for both clades, being higher in Clade I with 55% compared to Clade II with 49%. In contrast, the average protein length was strikingly similar in both clades, with median values of 338 and 339 for Clade I and Clade II, respectively (Figure 7D).

### Molecular signature of the thermophilic members of Anaerolineaceae

The phylogenomic tree also revealed that the thermophilic members of the family form a well-supported lineage. We aimed to identify the common proteins that are unique to the thermophile group. Through the analysis of orthologous groups of proteins, we identified a set of 22 proteins found exclusively in Anaerolineaceae thermophiles. These proteins were annotated as follows: phosphotriesterase, cation: proton antiporter, Na+/H+ antiporter subunits E and B, dual specificity protein phosphatase, VWA domain-containing protein, MoxR family ATPase, DUF4395 domain-containing protein, transcriptional repressor, Hsp20/alpha crystallin family protein, SufE family protein, AAA family ATPase, and the remaining were labeled as hypothetical proteins. These findings suggest a hypothesis highlighting potential proteins associated with thermal adaptation and response to oxidative stress in the thermophilic group. Notably, proteins like MoxR, known for its chaperone function, SufE, a metalloprotein involved in Fe-S cluster biogenesis, and the *mrp* operon encoding a bacterial Na+/H+ antiporter system, emerge as potential contributors to these adaptive mechanisms.

**FIGURE 8:**
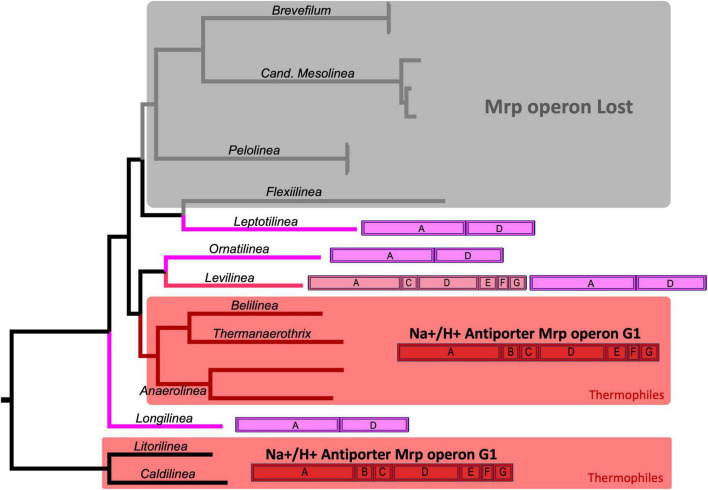
Evolution of the *mrp* Operon in Anaerolineaceae. The phylogenetic tree used in this illustration is the same as depicted in Figure 4. The *mrp* operon annotations for each genus showcase the arrangement of *mrp* subunits, with the coding sequences (CDSs) depicted in their actual order. The complete *mrpABCDEFG* operon (Group-1) is highlighted in red. The *mrpA’CDEFG* operon (Group-2) is represented in pink. The reduced *mrpAD* operon is depicted in violet. Light gray shading indicates genera where the operon appears to have been lost. Areas shaded in red represent thermophilic genera. As outgroups, Caldilineaceae members *Litorilinea* and *Caldilinea* are included.

Among this group of proteins, perhaps the most intriguing is the one annotated as Na+/H+ antiporter subunits. These proteins are encoded by a bacterial operon known as *mrp*, comprising 7 subunits from A to G. An evolutionary analysis of this operon revealed its complete presence, with all 7 subunits, exclusively in the thermophilic members of Anaerolineaceae. In contrast, the operon is incomplete in other species of Clade I of Anaerolineaceae (Figure 8). *Ornatilinea* and *Longilinea* exhibit a reduced version of the operon with only subunits A and D, which are the longest of the operon. In *Levilinea*, a more complex scenario was observed, where two *mrp* operons were detected; one with subunits A+D and the other missing only subunit B (*mrp* operon subunits A’CDEFG). For species in Anaerolineaceae Clade II, a distinctly different setup was noted. It appears that the *mrp* operon is lost in almost all its members, with proteins observed only in *Leptolinea*, which, however, harbors the reduced version of the operon with the A+D subunits.

## Discussion

The phylum Chloroflexota constitutes a highly diverse and deeply branching lineage of bacteria. Currently, it is categorized into nine classes (Kochetkova et al., 2020; Yan et al., 2020; Palmer et al., 2023): Anaerolineae, Ardenticatenia, Caldilineae, Chloroflexia, Dehalococcoidia, Ktedonobacteria, Thermoflexia, Thermomicrobia, and a class-level SAR202 cluster of uncultured bacteria. Members of the Anaerolineaceae family exhibit a broad distribution in anaerobic environments, including marine sediments, deep hot aquifers, soils, and anaerobic digesters. Notably, this family has been frequently identified in full-scale anaerobic digesters, where it plays a crucial role in the fermentation of carbohydrates and proteinaceous material (Petriglieri et al., 2018).

Resolving the evolutionary history of the Anaerolineaceae family has proven to be a challenging task, with previous publications presenting conflicting evolutionary hypotheses and low-support phylogenies (Yamada et al., 2006, 2007; Podosokorskaya et al., 2013; Imachi et al., 2014; Sun et al., 2016; McIlroy et al., 2017). This study represents a significant advancement as, for the first time, the evolutionary history of reference members of this family has been resolved with confident support.

Anaerolineaceae members exhibit low genome conservation profiles, with only a small fraction of their chromosomes aligning among different genera of the family, typically below 5%. This suggests considerable evolutionary distances and underscores the challenge of constructing well-supported trees using a limited number of loci (McIlroy et al., 2017; Kochetkova et al., 2020). Additionally, the proteome’s poor conservation is reflected in a median proteome coverage of just 58%. However, when comparing within each genus, when at least two species were available, a higher degree of genome conservation was observed, except for *Anaerolinea*.

The genomic and proteomic conservation analysis highlights some interesting patterns. *Pelolinea submarina* strains are highly similar, sharing over 99.9% coverage and nucleotide identity.

The high degree of identity between the two *Pelolinea* accessions might suggest that they are different genome sequences for the same isolate, potentially representing redundant entries in the NCBI database.

*Brevefilum* strains show significant similarity, and differences in genome and proteome conservation may be attributed to variations in genome assembly size. On the other hand, *Anaerolinea* reference species strains exhibit marked differences, sharing less than 5% of their genome and only 65% of their proteome. This divergence within *Anaerolinea* may suggest substantial genomic and functional diversity within this genus.

The Anaerolineaceae family exhibits a diverse evolutionary history, with *Longilinea arvoryzae* identified as the most ancestral member. Isolated from rice paddy soil, this mesophilic bacterium possesses the largest genome in the family at 4.1 Mb (Yamada et al., 2007). The family then diverges into two major clades, revealing distinct genomic characteristics.

In Clade I, which includes both thermophiles and mesophiles, a monophyletic group emerges with thermophiles clustered together. This group consists of the genera *Anaerolinea*, *Bellilinea*, and *Thermanaerothrix*, all of which thrive in high-temperature environments.

Clade II, on the other hand, encompasses only mesophilic microorganisms and shows significant genomic differences compared to Clade I. Genomes in Clade II tend to be smaller with lower GC content ratios. The number of genes per genome is also reduced in Clade II, aligning with the observed differences in genome sizes. However, the average protein length remains nearly the same in both clades, suggesting that genome reduction in Clade II may be associated with gene losses, possibly through genome deletions observed in other bacteria (Hershberg et al., 2007; Koskiniemi et al., 2012; Bobay and Ochman, 2017; Baena et al., 2023). Given that many genomes in Clade II are metagenome-assembled genomes (MAGs), future confirmatory analyses are crucial to mitigate potential effects of incomplete genomes on statistical analyses.

Based on the integrative analysis of comparative genomics and the evolutionary patterns within the Anaerolineaceae family, a hypothesis emerges. It appears that in this family, there might be an evolutionary tendency in the most recent clades toward smaller genome sizes with lower GC content, potentially linked to the adaptation of bacteria to a mesophilic lifestyle.

The relationship between GC content and growth temperature in bacteria has been documented in previous studies (Hu et al., 2022). Specifically, in the case of the bacterium *Tepiditoga spiralis* gen. nov., sp. nov., its lower GC content has been linked to an adaptation to a mesophilic lifestyle (Mori et al., 2020).

This hypothesis gains support from the observation that the closely related Caldilineaceae family is described as thermophilic. In Anaerolineaceae, some members in Clade I have retained their thermophilic physiology, but most of the family exhibits characteristics of mesophiles. This suggests a transition in the family’s evolutionary trajectory, highlighting the dynamic nature of microbial adaptation to environmental niches. Further research and confirmatory analyses are essential to validate and refine this hypothesis.

In our exploration of genomic features shedding light on adaptations to a thermophilic lifestyle, we identified 22 orthologous proteins exclusive to thermophilic Anaerolineaceae, absent in mesophiles. While some were annotated as hypothetical proteins, others offered clues to heat stress adaptations. For instance, the Hsp20/alpha crystallin family protein belongs to a group of heat shock proteins known as the sHSP (small heat shock proteins) involved in correct protein folding and avoidance of protein aggregation, particularly under stressful environmental conditions (Hartl et al., 2011; Lee et al., 2016). Additional proteins in the Anaerolineaceae proteome were also annotated as sHSP20. However, the one predicted to be exclusively present in Anaerolineaceae thermophiles is encoded by a gene found in a cluster of other copies with similar annotations.

One pair of proteins present exclusively in Anaerolineaceae thermophiles consists of MoxR and VWA proteins, encoded by an operon found in other bacterial taxa. MoxR proteins, described as AAA+ ATPases, may function as chaperones, participating in protein folding and maturation (Snider and Houry, 2006; Wong et al., 2014). In *Francisella tularensis*, a MoxR ATPase was associated with pH and oxidative stress resistance (Dieppedale et al., 2011).

A SufE orthologous protein was identified exclusively in the thermophiles of Anaerolineaceae. SufE is a metalloprotein involved in Fe-S cluster biogenesis, particularly under oxidative stress conditions (Blahut et al., 2020). Heat shock stress has been linked to oxidative stress in protozoan parasites (Alzate et al., 2007) as well as bacteria (Marcén et al., 2017), suggesting a potential role in higher oxidative stress resistance in Anaerolineaceae thermophiles.

Another intriguing discovery pertains to the evolution of the *mrp* operon within Anaerolineaceae. This operon, found widely in bacteria, exhibits a notably distinct structure across various bacterial clades. The *mrp* operon encodes a bacterial Na+/H+ antiporter system crucial for stress response, playing a vital role in maintaining intracellular pH, ion homeostasis, and cell survival under diverse environmental stress conditions (Ito et al., 2017). The *mrp* complexes and their subunits are anticipated to have undiscovered functions (Ito et al., 2017; Jasso-Chávez et al., 2017).

The *mrp* operon structure is diverse and has been categorized into three groups based on subunit content and gene order: Group-1, Group-2, and Group-3. The Group-1 *mrp* operon comprises seven genes consistently organized in the same order of subunits: A, B, C, D, E, F, G. Subunits A and D, the largest ones, are homologous to the respiratory chain complex I embedded in the cellular membrane. Group-2 mirrors the structure of *mrp* Group-1, but the first coding sequence (CDS) results from the fusion of genes for subunits A and B, termed *mrpA’*. The complete operon structure is *mrpA’CDEFG*. Group-3 consists of nine genes encoding the subunits, with irregular order (Ito et al., 2017).

We observed Group-1 and Group-2 *mrp* operons exclusively in Clade I of Anaerolineaceae. In Clade II, the operon appeared to be largely absent, except for *Leptolinea*, which possessed a reduced version of the operon containing only subunits A and D. Notably, only the thermophiles within the family carried the complete set of seven genes in the operon (Group-1 type, subunits A-G). Among the mesophiles in Clade I, all of them exhibited the reduced version of the operon, *mrpAD*, with the exception of *Levilinea*, which had two different versions: *mrpAD* and *mrpA’CDEFG*.

A noteworthy complementary finding is that the closely related family Caldilinecea, described as thermophilic, also harbors the Group-1 version of the operon, *mrpABDCEFG*, similar to the thermophilic members of Anaerolineaceae.

In the context of Anaerolineaceae thermophiles, the presence of the complete *mrpABCDEFG* Group-1 operon suggests a potential adaptation strategy to cope with the stress associated with elevated temperatures. This observation aligns with findings in other organisms, such as starving yeast exposed to thermal stress, where a transient drop in intracellular pH triggers the heat shock response (Gonçalves et al., 2020). Additionally, in *B. cereus* ATCC 14579, cells grown at low pH (5.5) exhibited acid stress adaptation, indicating a potential cross-adaptation between thermotolerance and acidotolerance (Rezkallah et al., 2016).

Finally, our phylogenomic analysis revealed a novel lineage within Anaerolineaceae Clade II. This clade is exclusively populated by metagenome-assembled genomes (MAGs) obtained from mesophilic wastewater treatment systems across different continents, including South America, North America, and Asia. Notably, half of these MAGs originate from industrial wastewater treatment systems in the Andean region of Colombia, where they act as dominant microbes in anaerobic digesters. Anaerolineaceae Clade II comprises mesophilic microorganisms belonging to the genera *Flexilinea*, *Leptolinea*, *Pelolinea*, *Brevefilum*, and a proposed *Candidatus* genus, *Mesolinea*. The *Candidatus* genus *Mesolinea* represents a novel lineage within Anaerolineaceae. The apparent loss of genes associated with heat stress tolerance, such as chaperones, oxidative stress response machinery, and the *mrp* operon, may signify an evolutionary adaptation of this lineage to a mesophilic lifestyle. In environments where the growth temperature falls below 40°C, some of the proteins involved in stress response, such as sHSP, MoxR, and Mrp, could be unnecessary or non-functional. Therefore, we propose the name *Candidatus Mesolinea* to denote its adaptation to a mesophilic lifestyle, considering that several of its ancestors exhibit a thermophilic physiology. It is likely that *Candidatus Mesolinea*, akin to other members of the Anaerolineaceae family, plays a pivotal role in the fermentation of organic material within anaerobic mesophilic environments, notably in anaerobic digesters, where they constitute one of the predominant taxonomic groups (Bovio-Winkler et al., 2021).

### Description of *Candidatus Mesolinea* gen. nov

*Candidatus Mesolinea* (Me.so.li’.ne.a. Gr. pref. mésos moderate; L. fem. n. linea, line; N.L. fem. n. *Mesolinea* line-shaped organism belonging to Anaerolineaceae family living in anaerobic mesophilic environments).

*Candidatus Mesolinea* gen. nov. is proposed based on its discovery in mesophilic anaerobic digesters from municipal wastewater treatment plants, alongside the absence of specific genes associated with the thermophilic lifestyle observed in other family members. Phylogenomic analysis supports that metagenome-assembled genomes (MAGs) attributed to this taxon constitute an independent lineage, comparable to previously described genera within the Anaerolineaceae family. The following MAGs are affiliated with this genus: GCA_002417685, GCA_008635265, GCA_034430095, GCA_937858405, GCA_003499715, GCA_003445715, and SAMD00738252.

### Description of *Candidatus Mesolinea colombiensis* sp. nov

*Candidatus Mesolinea colombiensis* (ko-lom-bi-EN-sis. L. fem. adj. gent.–of Colombia). The word “colombiensis” is a Latinized form derived from “Colombia,” the location where the species was discovered. In Latin, the suffix “-ensis” is commonly used to indicate origin or association with a particular place. Therefore, “colombiensis” means “of Colombia.”

*Candidatus Mesolinea colombiensis* gen. nov., sp. nov. represents the inaugural bacterium within this newly proposed genus, distinguished from its Anaerolineaceae family counterparts through meticulous phylogenomic analysis. This new taxon is delineated by a high-quality metagenome-assembled genome (MAG) that has a sequencing coverage of 229×, with completeness and contamination ratios of 92.79 and 1.44%, respectively. *Mesolinea colombiensis* gen. nov., sp. nov. thrives within the anaerobic digester of the Cañaveralejo WWTP in Cali, Colombia. Its genomic DNA was extracted from fresh biosolid samples as part of a metagenomic shotgun experiment. The MAG has been deposited in the DDBJ database under Bioproject PRJDB17532, with Biosample SAMD00738252.

## Data availability statement

The datasets presented in this study can be found in online repositories. The names of the repository/repositories and accession number(s) can be found below: https://www.ncbi.nlm.nih.gov/, PRJNA603294 and https://www.ncbi.nlm.nih.gov/, PRJNA1046455.

## Author contributions

KB-U: Data curation, Formal Analysis, Investigation, Methodology, Validation, Writing – original draft, Writing – review & editing. JA: Data curation, Formal Analysis, Investigation, Methodology, Project administration, Supervision, Validation, Visualization, Writing – original draft, Writing – review & editing.

## Footnotes

1
https://dfast.ddbj.nig.ac.jp

2
https://github.com/chriscreevey/catsequences
